# Development of a Self-Report Measure of Prediction in Daily Life: The Prediction-Related Experiences Questionnaire

**DOI:** 10.1007/s10803-024-06379-2

**Published:** 2024-05-07

**Authors:** Amanda M. O’Brien, Toni A. May, Kristin L. K. Koskey, Lindsay Bungert, Annie Cardinaux, Jonathan Cannon, Isaac N. Treves, Anila M. D’Mello, Robert M. Joseph, Cindy Li, Sidney Diamond, John D. E. Gabrieli, Pawan Sinha

**Affiliations:** 1 Program in Speech and Hearing Bioscience and Technology, Harvard University, Cambridge, MA USA; 2 McGovern Institute for Brain Research, Massachusetts Institute of Technology, Cambridge, MA USA; 3 Hock E. Tan and K. Lisa Yang Center for Autism Research, Massachusetts Institute of Technology, Cambridge, MA USA; 4 School of Education, Drexel University, Philadelphia, PA USA; 5 Department of Brain and Cognitive Sciences, Massachusetts Institute of Technology, Cambridge, MA USA; 6 Department of Anatomy and Neurobiology, Boston University School of Medicine, Boston, MA USA; 7 Present Address: The Donald and Barbara Zucker School of Medicine, Hofstra University, Long Island, NY USA; 8 Present Address: Department of Psychology, Neuroscience, and Behaviour, McMaster University, Hamilton, Ontario Canada; 9 Present Address: Department of Psychiatry and O’Donnell Brain Institute, UT Southwestern Medical Center, Dallas, TX USA

**Keywords:** Autism, Prediction, Self-report, Adults, Questionnaire

## Abstract

**Purpose:**

Predictions are complex, multisensory, and dynamic processes involving real-time adjustments based on environmental inputs. Disruptions to prediction abilities have been proposed to underlie characteristics associated with autism. While there is substantial empirical literature related to prediction, the field lacks a self-assessment measure of prediction skills related to daily tasks. Such a measure would be useful to better understand the nature of day-to-day prediction-related activities and characterize these abilities in individuals who struggle with prediction.

**Methods:**

An interdisciplinary mixed-methods approach was utilized to develop and validate a self-report questionnaire of prediction skills for adults, the *Prediction-Related Experiences Questionnaire* (*PRE-Q*). Two rounds of online field testing were completed in samples of autistic and neurotypical (NT) adults. Qualitative feedback from a subset of these participants regarding question content and quality was integrated and Rasch modeling of the item responses was applied.

**Results:**

The final *PRE-Q* includes 19 items across 3 domains (Sensory, Motor, Social), with evidence supporting the validity of the measure’s 4-point response categories, internal structure, and relationship to other outcome measures associated with prediction. Consistent with models of prediction challenges in autism, autistic participants indicated more prediction-related difficulties than the NT group.

**Conclusions:**

This study provides evidence for the validity of a novel self-report questionnaire designed to measure the day-to-day prediction skills of autistic and non-autistic adults. Future research should focus on characterizing the relationship between the *PRE-Q* and lab-based measures of prediction, and understanding how the *PRE-Q* may be used to identify potential areas for clinical supports for individuals with prediction-related challenges.

The ability to make, utilize, and learn from predictions broadly impacts our engagement with the world. Colloquially, the term prediction is used to describe expectations of what will happen next. People make and use predictions every day, often with little conscious effort. For example, when catching a ball, we predict the ball’s trajectory and adjust the position of our hands to anticipate its arrival. Predictions are more than simple estimates about the future; they reflect complex, multisensory, and dynamic processes, which are continually adjusted in real time to reflect new information and occur throughout one’s daily life. Pellicano and Burr (2012) first proposed that prediction-related challenges may underlie characteristics associated with autism. Since then, additional researchers have put forth similar frameworks (Lawson et al., 2014; Sinha et al., 2014; van de Cruys et al., 2014). However, no studies to date have characterized self-reported difficulties with prediction in autistic or non-autistic adults.

## Defining Prediction

Researchers have attempted to operationalize and study the construct of prediction through the development and refinement of the *predictive coding framework*. The predictive coding framework posits that a primary function of the brain is to integrate prior knowledge and current context to continuously generate predictions in order to efficiently process streams of incoming information (Friston, 2005, 2010; Huang & Rao, 2011; Rao & Ballard, 1999; Spratling, 2017). This framework has been put forth as a unifying theory of brain function, and it provides a theoretical lens through which to investigate and understand the computational and neural mechanisms underlying various cognitive processes. Indeed, behavioral and imaging studies in the general adult population have provided supporting evidence for the predictive coding framework across many domains, including motor, vision, and language (Rauss et al., 2011; Schrimf et al., 2021; Shipp et al., 2013).

The operational definition of prediction and predictive coding varies across the literature on predictive processing. Generally, within the predictive coding framework, prediction is conceptualized as the use of one’s mental models (i.e., internal representations of causal relationships) to generate expectations of incoming sensory stimuli based on running estimates of hidden states and processes in the world. Differences between one’s expected outcome and the actual outcome lead to errors that are used to update one’s mental model and modify future predictions. In the current study, the operationalization of prediction put forth by Cannon and colleagues (2021) was adopted, as it aimed to integrate multiple proposed definitions of predictive processing. Figure S1 in the Supplemental Materials illustrates the definition of prediction (Cannon et al., 2021) as a process that (a) is based on a known association between an antecedent and consequence (*mental model*), (b) is generated in response to a given antecedent, context, and/or event (*inference*), (c) directly affects an individual’s neural or behavioral response in preparation of or upon the arrival of the consequence, or in future occurrences of the antecedent (*deploy prediction*), (d) may lead to surprise if the prediction is not aligned with the actual sensory-motor input (*surprise*), and (e) can lead to updates of the mental model, particularly following prediction errors or changes to the antecedent-consequence relationship over time (*dynamic update*).

## Atypical Prediction

Beyond prediction-related models of brain function in non-clinical populations, theoretical accounts have hypothesized that atypical prediction may underlie traits associated with autism as well as other clinical conditions such as schizophrenia and dementia. In the study of autism, multiple intersecting theories posit that atypical prediction may underlie key characteristics of the condition in children and adults (e.g., Lawson et al., 2014; Sinha et al., 2014; van de Cruys et al., 2014). Empirical evidence for prediction-related difficulties in autistic individuals^1^ is mixed and nuanced, with greatest support for prediction-related differences shown via reduced habituation and repetition suppression, reduced spontaneous predictive movement, reduced predictive eye gaze, difficulty with social predictions, and potential differences in the learning of predictive relationships (for two reviews, see Angeletos et al., 2023; Cannon et al., 2021). In autistic adults, the most consistent evidence for prediction-related differences included reduced habituation of brain response to repeating stimuli, particularly for faces (D’Mello et al., 2023; Ewbank et al., 2017; Tam et al., 2017), reduced anticipatory saccades in response to predictable visual stimuli (Schuwerk et al., 2016), and different trajectories of predictive relationships (Lawson et al., 2017). Similar theories of prediction-related difficulties have emerged for individuals with schizophrenia (Sterzer et al., 2019) and dementia (Kocagoncu et al., 2021). Further research is warranted to better understand the presence, extent, specificity, and impact of potential prediction-related differences within and across clinical populations.

## Existing Measures of Prediction in Autism

Substantial investigation of typical and atypical prediction and predictive coding has been conducted, with both children and adults, using a wide range of empirical measures, including both neuroimaging and behavioral (e.g., psychophysical) techniques. Here, existing measures within the autism adult literature are highlighted given their relevance to the current study, although many of the measures described have also been implemented with autistic children. Neuroimaging has elucidated several underlying differences in the neural correlates of prediction. Researchers have used electroencephalography (EEG) and functional magnetic resonance imaging (fMRI) to study how individuals habituate and adapt to predictable visual or auditory sequences (D’Mello et al., 2023; Ewbank et al., 2017; Ruiz-Martinez et al., 2020); EEG to understand surprise responses to unexpected deviations in auditory (including linguistic) or visual stimuli (Barzy et al., 2020; Goris et al., 2018); EEG to understand brain-based ramping activity preceding predictable targets (Thillay et al., 2016); and fMRI to localize and quantify prediction-related brain activation (Balsters et al., 2017). Beyond neuroimaging approaches, researchers have used eye tracking to study how individuals visually anticipate upcoming actions (Ganglmayer et al., 2020). Pupillometry (i.e., pupil dilation measurement) has also been used as a physiological measure of surprise in response to expected and unexpected consequences (Lawson et al., 2017). Behaviorally, researchers have employed statistical learning tasks to assess association learning between basic and complex antecedents and consequents. These tasks have been used to understand processes in autistic adults such as how individuals utilize context to make predictions (Rybicki et al., 2021; Treves et al., 2023, as well how individuals use predictive information to respond more quickly to predictable targets (Cannon et al., 2023). Finally, some investigations utilize self-report measures that ask participants whether a given outcome was expected or not, thus obtaining a report of surprise related to prediction (Balsters et al., 2017; Sheppard et al., 2016).

The wide range of existing experimental measures for characterizing prediction have several limitations. First, measures of prediction typically investigate a single predictive process, domain, or phenomenon (e.g., visual, motor, auditory). Indeed, reviews of prediction-related tasks in the field of autism (Cannon et al., 2021; Merchie & Gomot, 2023) show a trend of testing a single behavioral or neural measure, as opposed to multiple measures that together may better reflect the multifaceted phenomenon of prediction. There are currently few behavioral and no self-report measures that incorporate the multiple modalities that make up the complex phenomenon of prediction. Second, research and experimental measures (e.g., neuroimaging techniques) typically require an in-person visit to a research laboratory, which can limit the number and diversity of individuals who participate in such studies. Finally, the tools available to measure typical and atypical prediction and predictive processing lack a connection to individuals’ daily experiences. Thus, it is often unclear what level (if any) of functional impact is experienced by individuals who demonstrate difficulty with isolated prediction-related empirical tasks.

## Need for a Self-Report Measure of Prediction

In clinical and non-clinical adult populations alike, a self-report measure of prediction skills and challenges would improve these individuals’ understanding of daily experiences and difficulties in relation to predictive processing frameworks. Such a measure could quantify to what extent and in which contexts people experience prediction-related difficulties across multiple domains (e.g., motor, social, sensory) that make up the complex phenomenon of prediction. Implementation of such a measure of prediction-related daily living skills could also advance novel translational understanding of quality of life and independence. This type of self-report measure could be correlated and validated with empirical prediction tasks (e.g., eye-tracking, brain-based, and behavioral), to better understand the relationship between these tasks and daily challenges, guide further naturalistic experimental design, and potentially influence the development of more effective clinical supports and interventions. A validated self-report measure can be administered remotely, reducing time and effort for individuals being assessed and increasing inclusion of participants who may be unable to attend in-person visits (e.g., due to transportation limitations, employment, familial responsibilities), or when in-person laboratory visits are prohibited, as was the case during the COVID-19 pandemic.

A self-report measure that has been validated with both neurotypical (NT) and autistic adults (versus validated in NT adults or autistic adults alone) would prove useful in fully capturing a wide range of prediction skills, given the proposed prediction challenges in autism specifically. The results from this measure could improve identification of daily challenges and potentially lead to enhanced support, treatment targets, and environmental modifications for autistic individuals.

## Study Aim

The purpose of this study was to expand understanding of predictive processing through the development of a survey instrument called *The Prediction-Related Experiences Questionnaire (PRE-Q;* Bungert et al., 2024) designed to align with Cannon et al.’s (2021) model of predictive processing. An intended aim of the *PRE-Q* is to assess NT and autistic adults’ self-perceptions of their prediction skills in activities of daily life to identify strengths and challenges in predictive processing. Five research questions were addressed in the validation process using the Rasch measurement model (1960/1980) and traditional inferential statistics to achieve a parsimonious instrument and inform the validity evidences for response process, internal structure, and relationship to other variables (American Educational Research Association [AERA] et al., 2014).

### Response Process Validity Evidence

1. How are the properties of the 4-point response category functioning for the *PRE-Q*?

### Internal Structure Validity Evidence

2. To what extent is the *PRE-Q* unidimensional (item fit, internal consistency)?
3. What measurement redundancies exist in the initial *PRE-Q* that can be removed to form a more parsimonious measure of self-reported predictive processing?

### Relationship to Other Variables

4. Did NT and autistic adults significantly differ in their *PRE-Q* logit scores?
5. Was there a significant correlation between *PRE-Q* logit scores and behavioral scores on prediction-related tasks for a sub-sample of NT and autistic adults?

## Methods

**Fig. 1:**
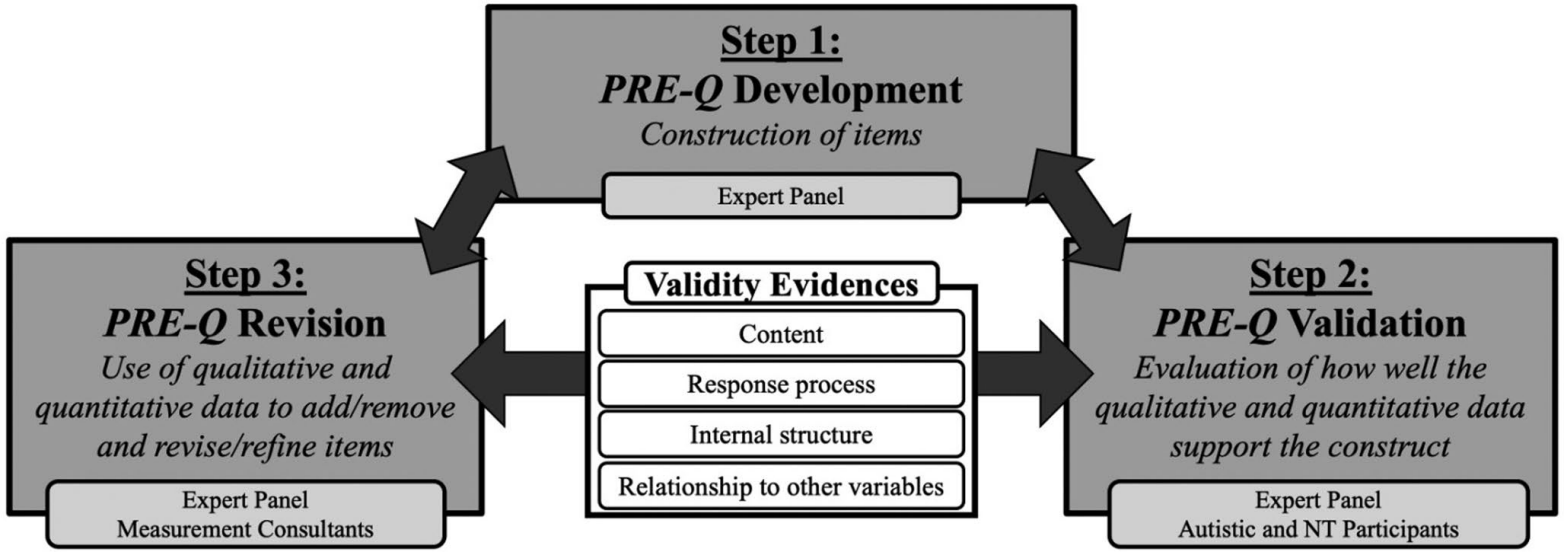
Visualization of the three iterative stages of the PRE-Q Development. Note: NT = Neurotypical. The light gray boxes indicate the people who were involved during each stage of *PRE-Q* development

Two methodological frameworks guided the instrument development in this study. Luyt’s (2012) iterative framework for measurement development, validation, and revision was used in implementing a design-based approach. This framework consisted of three cyclical, interconnected steps of instrument development and validation (Fig. 1). Step 1 was measurement development, which involved defining the construct and developing indicators (i.e., items) that operationalized the construct. Step 2 was measurement validation, which involved evaluating to what extent the quantitative scores and/or qualitative data collected supported meaningful interpretation of the construct. Step 3 was measurement revision whereby quantitative and qualitative data were used to inform decisions related to instrument revisions. This study was not preregistered.

Additionally, the *Standards for Educational and Psychological Assessment* (AERA et al., 2014) was used as a framework for collecting data and evaluating validity evidence of the *PRE-Q*. Through the *Standards*, instrument developers are encouraged to employ a mixed methods approach to instrument construction and collect multiple types of validity evidence to provide more robust support for inferences drawn from the measure (Luyt, 2012; Koskey et al., 2018; Onwuegbuzie et al., 2010). Five forms of validity evidence are described in the *Standards*: content (item alignment with construct); response process (participant understanding of instrument as researchers intended); consequences of testing (potential negative impact on participants or bias); internal structure (unidimensional and reliable measures); and relationship to other variables (alignment of instrument outcomes with other hypothesized variables).

The measurement development process is outlined below, including the initial item pool informed by Cannon and colleagues’ (2021) definition of prediction, an expert panel, and feedback from a group of autistic adults. Following the initial item pool development, the quantitative field-testing procedure is outlined including two rounds of data collection. Measurement revisions based on qualitative and quantitative field-testing findings are described throughout this iterative process.

### Initial Measurement Development

#### Expert Panel Item Construction

A panel of ten experts comprising researchers and clinicians was formed to construct the initial item pool. The item development panel team consisted of researchers collaborating on a project related to prediction, and included nine members from the same location and one collaborator from a different institution in the same city. Table S1 in the Supplemental Materials lists the panel members’ relevant expertise. All panel members were actively involved in research projects related to prediction and autism at the time of their involvement in the *PRE-Q’s* development. Additionally, two consultants from another institution and city provided guidance on the survey development methodology and analyses, but did not directly participate in the item construction panel. Items were constructed to align with Cannon et al.’s (2021) definition of predictive processing to inform content validity evidence. Panel members were instructed to write items that: (a) were appropriate for the intended population of autistic and NT adults; (b) targeted behaviors, thoughts, or feelings representing prediction-related sensory, social, motor, and daily living experiences across the four phases of predictive processing; and (c) included easier to harder predictions to reflect a range of difficulty levels. A total of 88 items were constructed.

#### Expert Rating of Alignment With Prediction Construct and Prediction Phases

Panel members rated each of the 88 items in two steps: First, based on how well each item aligned with the construct of prediction and second, based on how well each item aligned with the phases of prediction (Inference, Deployment, Surprise, or Dynamic Update). Panel members also provided qualitative feedback on the clarity of item wording at this stage. Based on the ratings of alignment with construct, 37 items (42.05%) were removed. Based on the ratings of alignment with prediction phases, seven items were removed. Following the removal of these items, an additional 13 items were created related to the phase of Surprise and the Sensory domain to balance the distribution of items across phases and domains. Additional information about the rating processes is included in the Supplemental Methods.

#### Participant Feedback

Participant feedback informed response process validity evidence in two ways. Participants who provided feedback were recruited as described in “Quantitative Sampling Procedures and Participants” below. First, when participants completed the self-report prediction items, they were asked to indicate if they “did not understand” any of the existing items and were allowed to write a short description of what was confusing about the item. Second, a separate group of 15 autistic adults (who had not been asked to complete the self-report items) were recruited to complete an online survey consisting of seven open-ended questions related to prediction-related challenges they might have encountered. Two questions asked what predictions these autistic individuals make when “listening to or watching something” and when “tasting or smelling something.” An example response was, “I might make predictions before I taste or something and this would be whether I expect to like it or not.” The next four items asked to describe when these individuals are surprised during daily life, social situations, while sensing (listening, watching, touching, tasting, or smelling) something, and participating in a physical activity. An example response was, “If an appliance breaks when I’m about to use it when it was fine the day before.” A final item asked what things are hardest to predict. An example response was, “People’s behaviors/reactions, especially if they are more acquaintances to me than friends.”

The open-ended question feedback was used to guide construction of additional items targeting the Sensory domain and the Surprise phase to improve content validity. Two members of the panel met to convert autistic adults’ responses into new prediction items. For example, the response “I sometimes make predictions as to what will happen next if I am watching a movie or TV show,” was converted into the item “When watching television or a movie, I can easily tell what a character will say next.” A total of 24 items were developed based on the responses to yield an 81-item *PRE-Q* for quantitative field-testing.

### Quantitative Sampling Procedures and Participants

To address research questions 1−4, participants were recruited through online platforms to complete the *PRE-Q* (initial 81-item or refined 19-item versions), as well as additional phenotypic characterization measures. To address research question 5, a subset of autistic and NT individuals completed a behavioral auditory motor synchronization task of predictive processing, (part of a larger online study) in addition to the refined 19-item *PRE-Q*.

### Participants

Autistic participants were recruited through “SPARK” (Simons Powering Autism Research), a national database of individuals who have an existing clinical diagnosis of autism with a high degree of diagnostic validity (Fombonne et al., 2022; SPARK Consortium, 2018). NT participants were recruited through Prolific (Prolific.org), an online scientific research study adult recruitment tool. The initial 81-item *PRE-Q* was completed by a total of 193 participants, including 40.93% autistic (*n* = 79; 42 females; *M*_age_ =27.5 yrs. ± 5.2 yrs.) and 59.07% NT (*n* = 114; 42 females; *M*_age_ =30.1 yrs. ± 7.2 yrs.) individuals.

The refined 19-item *PRE-Q* was completed by 141 non-overlapping participants including 50.35% autistic (*n* = 71; 26 females; *M*_age_ = 29.6 yrs. ± 8.7 yrs.) and 49.65% NT (*n* = 70; 35 females; *M*_age_ = 29.7 yrs. ± 7.2 yrs.) individuals. Race and ethnicity data were not collected. A subset of participants completed the auditory motor synchronization task. Forty-three of these participants were eligible to be included in task-specific behavioral performance analyses using data quality inclusion criteria established by Cannon et al. (2023). This sub-sample was comprised of 35% autistic (*n* = 17; 11 female) and 64% (*n* = 26; 13 female) NT individuals. In addition to sex assigned at birth (reported above), gender identity was also collected for this sub-sample of participants (Autistic participants’ gender identities: 6 female, 7 male; 2 nonbinary; NT participants’ gender identities: 12 female, 13 male; 1 nonbinary).

### Measures

#### PRE-Q

The *PRE-Q* was intended to measure an individual’s perception of their predictive processing aligned to the Cannon et al. (2021) theoretical model. It was designed for adult populations including autistic and NT individuals who can read and respond independently to simple one-sentence statements. An initial 81-item *PRE-Q* for quantitative field-testing consisted of items representing the four phases of Cannon et al.’s (2021) proposed model of predictive processing: Inference (27 items), Deployment (27 items), Surprise (17 items), and Dynamic Updating (10 items). Across these four phases, items presented predictive processing contexts related to the four domains of Motor (19 items), Sensory (14 items), Social (32 items), and Daily Living (16 items). Items were rated on a 4-point scale (0 = “Not at all like me,” 1 = “Not like me,” 2 = “Like me,” and 3 = “Just like me”). Higher ratings indicated a higher level of prediction skills for 63 items and lower prediction skills for 18 items that were reverse coded for scores to be interpreted in the same direction. An example reverse coded item was, “If I pop a balloon, I am startled by the noise.” The Flesch-Kincaid grade level score for the 81-item *PRE-Q* was 5.8, at the targeted fifth-grade level (Good Calculators, 2023).

A refined, parsimonious 19-item *PRE-Q* for quantitative field-testing consisted of items representing the four domains of the theoretical model of predictive processing: Inference (4 items), Deployment (8 items), Surprise (8 items), and Dynamic Updating (2 items). Across these four phases, items presented predictive processing contexts related to Motor (7 items), Sensory (6 items), and Social/Daily Living (6 items). Social and Daily Living were collapsed when the expert panel determined that the remaining Daily Living items were all related to social interactions. Items were rated on the same 4-point scale as the initial instrument. Higher ratings indicated a higher level of predictive processing for 15 items and lower predictive processing for 4 items that were reverse scored for scores to be interpreted in the same direction. Flesch-Kincaid grade level score for the 19-item *PRE-Q* was 5.9, at the targeted fifth-grade level. This final instrument is available online for use by researchers and clinicians (Bungert et al., 2024) and items are detailed in the Supplement (Table S2).

#### Predictive Processing Behavioral Tasks

Two prediction-related online tasks were used as criteria to correlate with the sub-sample of participants’ *PRE-Q* scores. In the first task, participants listened to 110 short metronomes consisting of 5, 6, or 7 beeps with a 700ms inter-beep interval in which the last interval was perturbed in time (-150ms, -100ms, -60ms, -30ms, -15ms, 0ms, + 15ms, + 30ms, + 60ms, + 100ms, + 150ms; negative numbers represent early beeps and positive numbers indicate late beeps). Metronomes were presented in a pseudorandom order. Participants were instructed to judge whether the last tone in each sequence was early or late. Their fraction of early/late responses as a function of shift size was fit with a logistic function, and the slope parameter was used as a measure of timing sensitivity. We interpreted the slope of perception following perturbations as a measure of ability to make precise auditory predictions based on rhythmic context (Stage 2 discussed in “Defining Prediction” above).

In the second task, participants heard nine sequences of beeps based on a steady metronome with 700ms inter-beep interval, with occasional timing perturbations (-50ms, -25ms, + 25ms, + 50ms; negative numbers represent early beeps and positive numbers indicate late beeps). Participants were instructed to tap along to the beeps on their laptop outside of the trackpad and keyboard area. From this task, we extracted two measures related to prediction (the dependent variables): tapping imprecision and tapping correction. Tapping imprecision refers to the timing difference between the participant’s response and the target sound. Tapping correction refers to the change in tap response timing after the participant hears a temporal deviation a in the regular beep sequence. For the timing tapping imprecision, we calculated the log of the standard deviation of the signed delay between tap and beep. We interpreted the magnitude of the standard deviation as a measure of ability to deploy and update predictive models (Stage 3 and Stage 4 discussed in “Defining Prediction” above). For the tapping correction measure (Phase Correction Responses), we calculated the magnitude of the correction of tap timing following perturbations (see Supplemental Information for details), and interpreted it as a measure of ability to dynamically update predictive models (Stage 4 discussed in “Defining Prediction” above). This task and its derived measures have been used previously to quantify temporal prediction-related abilities in autistic and NT adults (Cannon et al., 2023; Edey et al., 2019; Morimoto et al., 2018; Repp & Su, 2013; Vishne et al., 2021). Tapping was recorded and reviewed for quality (see Supplemental Information for details).

### Data Analysis

Rasch (1960/1980) measurement was employed in the quantitative field-testing component of this study as its effectiveness has been demonstrated in survey development, refinement, and validation research (see Bond & Fox, 2015; Boone et al., 2011; Koskey & Stewart, 2014; Liu, 2010; Wright, 1996). Numerous advantages are commonly cited for use of the Rasch model (1960/1980) over Classical Test Theory (CTT) approaches for instrument construction; Three are highlighted here (for additional advantages, see Andrich, 2011 and Bond & Fox, 2015). First, an overall person ability score is computed and transformed into log-odd units (i.e., logits) along the linear measure with item mean set at 0 logits. Placing persons and items on a common scale provides for more meaningful interpretation of person ability along the latent construct. Second, a more robust evaluation of the rating scale properties is facilitated through rating scale functioning analysis examining for monotonicity of the scale and explained in greater detail below. Third, because Rasch is a probabilistic model, it can handle missing data by estimating missing responses. With CTT, each survey item is assumed to possess the same amount of the trait being measured regardless of how challenging to endorse. Thus, if two participants *strongly agreed* with seven different items on a 10-item survey while leaving the remaining 3-items unanswered, they would receive the same score under a CTT model. Rasch measurement can, however, differentiate between these two individuals who endorsed a similar number of items with varying difficulty levels by giving the participant who endorsed more challenging items a higher measure compared to the individual endorsing easier items. Winsteps version 4.8.0 (Linacre, 2021) software was utilized for all Rasch analyses in this research. Additional details regarding Rasch measurement are included in the Supplemental Methods.

### Response Process Validity Evidence

Linacre (2002a) established guidelines for optimizing rating scales to ensure strong measure stability, measure accuracy (fit), description of the sample, and inferences for the next sample. While all guidelines are not required in all situations (Linacre, 2002a), some are essential and appropriate for this study. As such, four key optimization guidelines were investigated to inform whether respondents were using the scale categories as researchers intended.

1. ***A minimum of 10 observations in each category*** is essential for measuring stability and helpful for measuring accuracy (fit) and inference for next sample. Low frequency of observations in any category can result in poorly estimated or unstable step calibrations.
2. ***Observed average person measures advance with categories*** is essential for measure accuracy (fit), description of the sample, and inference for next sample. It is also helpful for measuring stability. When this guideline is met, it demonstrates that higher categories indeed reflect higher measures.
3. ***Outfit mean-squares (MNSQ) < 2.00*** is essential for measure accuracy (fit) and helpful for measure stability, description of the sample, and inference for the next sample. When a category’s MNSQ is > 2.00 more unexpected randomness is present than the amount expected (1.00).
4. ***Step calibrations advance*** is helpful for inferences related to the next sample. When step calibrations do not advance, it suggests that categories are disordered and as one advances along the variable a category may not be observed or necessary.

### Internal Structure Validity Evidence

While unidimensionality of a construct is a measurement specification regardless of model used, concrete criteria for assessing dimensionality do not exist and thus it cannot be determined in an either-or fashion (Smith, 2002). Rather, dimensionality is necessarily evaluated on a continuum of more to less unidimensional based on a holistic interpretation of findings from multiple psychometric indices. Rasch psychometric indices used in this study to examine unidimensionality of predictive processing as measured by the *PRE-Q* were: item fit statistics (infit, outfit, point-biserial correlation), measure consistency statistics (item and person reliability and separation), and item redundancy (logit-measures with *SEM* and variable map).

#### Item Fit Statistics

Rasch item infit, outfit, and point-biserial correlation indices provide information about unexpected patterns of responses and how well the data fit the measurement model. Item infit is related to patterns of item responses with difficulties closer to a person’s measure, while outfit is based on response patterns for items with difficulty measures further away from a person’s logit-score (missing easier items or correctly answering more difficult items) (Linacre, 2002b). According to Linacre (2002b) infit and outfit item mean-square (MNSQ) statistics between 0.50 and 1.50 suggest an item is functioning well and offering information that is productive for measurement. An item infit or outfit MNSQ below 0.50 or between 1.51 and 2.00 indicates an item is less productive for measurement but does not degrade the measure. However, an MNSQ above 2.00 is thought to distort the measure and should likely be removed.

An item’s point-biserial correlation is a representation of the item’s positive contribution to the overall measure. Items with a positive point-biserial are contributing positively to the measure, and items with a negative point-biserial are functioning in opposition to the construct (Wright, 1992). Thus, items with a negative point-biserial should be removed as they do not fit well with the measure’s meaning.

#### Measure Consistency Statistics

Rasch reliability for items and persons are used to investigate internal consistency of measures. Rasch separation statistics report the number of statistically different items or person groups that can be detected with a latent variable. Higher reliability and separation values represent stronger constructs capable of measuring wider ranges of the trait being studied. Duncan and colleagues (2003) have established Rasch reliability and separation criteria: unacceptable (reliability < 0.70; separation < 1.50), acceptable (reliability = 0.70–0.79; separation = 1.50–1.99), good (reliability = 0.80–0.89; separation = 2.00–2.99), and excellent (reliability ≥ 0.90; separation ≥ 3.00).

#### Item Redundancy

One purpose of this study was to reduce a larger item pool of predictive processing items on the *PRE-Q* to a more parsimonious data-informed set of items that maintained strong psychometrics. To empirically do this, a Wright map (or variable map) was examined along with item logit-measure difficulties and corresponding standard errors to determine statistical similarity. A Wright map presents person abilities on one side of a logit ruler and item difficulties on the other. Item hierarchy on a Wright map is established by participant responses with easier to endorse items at the bottom and more challenging to endorse items at the top (Bond & Fox, 2015). When statistical redundancy in item logit-measure was identified, the item writing team further inspected item content to determine which items should be retained and which should be removed.

### Relationship to Other Variables

Final *PRE-Q* 19-item person logit-measures were used to look for differences in self-reported prediction-related skills between NT and autistic adults. It was anticipated that there would be a significant difference in prediction skills by group, with autistic adults scoring significantly lower than NT adults. An independent samples *t*-test was implemented to address this hypothesis. Additionally, it was hypothesized that a significant relationship between final *PRE-Q* 19-item person logit-measures and outcome measures from the two behavioral tasks of prediction yielding three outcome measures (timing sensitivity, tapping imprecision, and tapping correction) would exist for a sub-sample of autistic and NT adults. To test this hypothesis, a Pearson correlation was conducted between participants’ *PRE-Q* logit scores and these outcome measures, for the full sample and for each diagnostic group separately, with the Bonferroni correction for multiple comparisons. Materials and code are available by emailing the corresponding author.

## Results

### Response Process Validity Evidence

Findings related to Rasch rating scale analysis for both the initial field-testing (81 items) and final field-testing (19 items) instruments were strong, as shown in Table 1. Regardless of field-testing trial, the rating scale performed well and in alignment with Linacre’s (2002a) criterial for optimizing rating scales. To summarize, more than 10 observations were noted for each scale category in initial (range 706–4,674 responses per category) and final (range 240–791 responses per category) field-testing trials. Average person logit measures progressed from lowest category (“Not at all like me”) to highest (“Just like me*”*), suggesting that participants with more of the latent trait were using higher scale categories compared to those with lower levels. All outfit mean squares were very close to the benchmark of 1.00 and none came close to exceeding 2.00, which implies an appropriate amount of randomness exists in the responses across rating scale categories and field-testing trials. Step calibrations advance monotonically from lowest scale category to highest indicating there is no redundancy in categories. Figure S2 in the supplemental materials demonstrates this graphically, as distinct hills were produced for the categories in each field-testing trial with the hills being even more pronounced in the final field-testing run compared to initial field-testing. Collectively, these findings imply the rating scale is working effectively to produce strong measure stability, measure accuracy (fit), provide appropriate description of the sample, and generate inferences for the next sample (Linacre, 2002a).

**Table 1:** PRE-Q rasch rating scale findings for initial and final field-testing trials

Guideline Scale Category	Initial Field-Testing (81 Items, 193 Participants)	Final Field-Testing (19 Items, 141 Participants)
**10 + Observations**	Frequency of Responses	
Not at all like me Not like me Like me Just like me	706 1,997 4,674 2,997	240 636 791 667
**Measures Advancement**	Average Person Measures in Logits	
Not at all like me Not like me Like me Just like me	−0.70 0.21 0.67 1.25	−0.88 −0.15 0.73 1.79
**Outfit MNSQ < 2.0**	Mean Square	
Not at all like me Not like me Like me Just like me	1.12 0.96 0.93 0.97	1.09 0.97 0.96 0.98
**Step Calibration Advancement**	Step Difficulty Measures in Logits	
Not at all like me Not like me Like me Just like me	None −1.01 −0.38 1.39	None −1.52 0.10 1.42

### Internal Structure Validity Evidence

Overall, various psychometric indices related to unidimensionality of the *PRE-Q* demonstrated the construct of predictive processing was being measured reasonably well with the initial 81-item survey and considerably better with the more parsimonious 19-item survey (see Table 2). In summary, person and item reliability remained somewhat similar between survey distributions with person reliability in a good range and item reliability being excellent. Item separation was similar across field-testing trials with nearly three distinct groups of people being measured. Further, item separation increased substantially with the shorter 19-item version (measuring seven distinct groups) compared to the longer 81-item survey (measuring approximately four distinct groups). Two items had negative point-biserial correlations from the 81-item initial field-testing trial, suggesting their removal was necessary as they did not fit in the construct. No items had a negative point-biserial correlation in the final 19-item survey, indicating all items were contributing positively to the measure. In the initial field-testing trial of 81-items, five items had an MNSQ infit or outfit that was less productive but not degrading, and one item had an outfit at a level that degraded the measure and required removal. Only one item had minor misfit (less productive, not degrading) among the 19-items from the final field-testing. Table S3 in the Supplemental Materials reports item statistics (logit-measure and *SEM*, infit and outfit MNSQ, point-biserial correlation) for the final 19-items sorted by difficulty logit measure. A detailed description of the item hierarchy is provided in the Discussion section.

**Table 2:** Psychometric properties of PRE-Q in each field-testing iteration

	Field-Testing Iteration	
	Initial	Final
Criteria and Guidelines^1^	81 Items / 193 Participants	19 Items / 141 Participants
**Reliability** (< 0.70 = Poor; 0.70 = Acceptable; 0.80 = Good; 0.90 = Excellent)		
Person	0.89	0.87
Item	0.93	0.98
**Separation** (< 1.5 = Poor; 1.5 = Acceptable; 2.0 = Good; 3.0 = Excellent)		
Person	2.82	2.59
Item	3.77	7.00
**Point-Biserial** (Positive pt-bis required)		
Items with negative pt-bis	2 *SE*, 2 SO	None
**Fit** (MNSQ > 2.0 = Degrades measure; <0.5 or > 1.5 = Less productive, not degrading; 0.5 to 1.5 = Productive)		
Items with less productive infit Items with less productive outfit Items with infit degrades measure Items with outfit degrades measure	3 SE, 4 SO 1 SE, 5 SO, 1 D None 1 *SE*	1 *SE*

Note. D = Daily Living, SO = Social, SE = Sensory, M = Motor

^1^Reliability and separation criteria and guidelines (Duncan et al., 2003); Item fit criteria and guidelines (Linacre, 2002b)

#### Item Redundancy

**Fig. 2:**
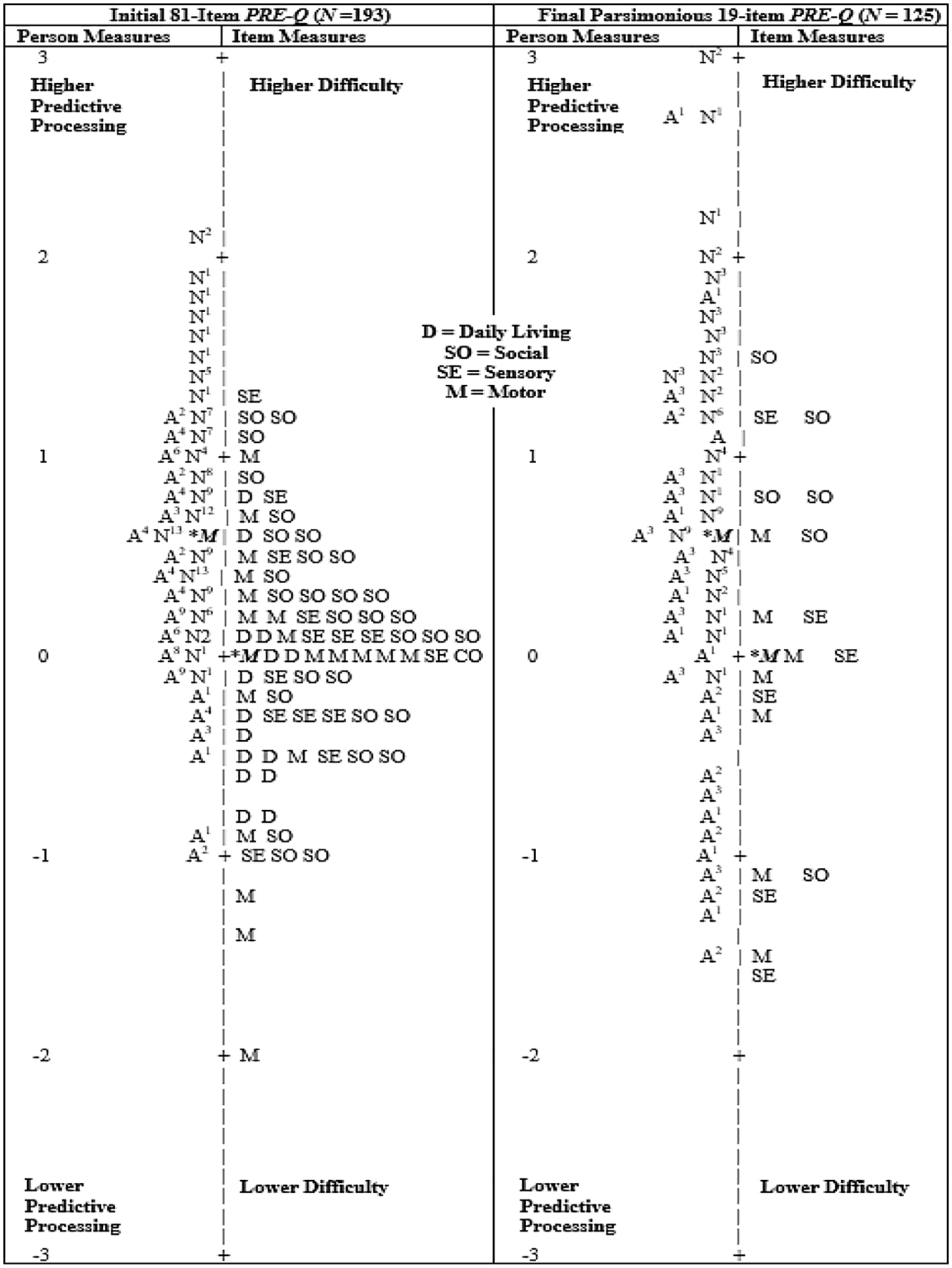
Initial and Final PRE-Q Item-Person Ordering Along the Continuum of Predictive Processing. Note: A = Autistic adult. N = Neurotypical adult. *M = mean person ability or item difficulty. Superscript indicates number of participants yielding that person measure (e.g., A^9^ = nine autistic participants)

Figure 2 displays two Wright maps side-by-side, illustrating the initial and final item-person ordering along the continuum of predictive processing. On the left is the initial 81-item *PRE-Q* Wright map and to the right is the final 19-item version. In each Wright map, participants are denoted on the left of their dashed line with either an A (Autistic) or N (Neurotypical) while items are on the right of their map’s dashed line (D = Daily Living, SO = Social, SE = Sensory, M = Motor). Items towards the top have higher difficulty logit-measures (more challenging to endorse) with participants self-reporting higher levels of predictive processing. Less difficult items to endorse are at the bottom of the maps along with participants who self-reported lower levels of predictive processing. For both maps, the mean (designated with an *M) of participants is above the item mean indicating that the average person finds most of the predictive processing items generally easy to endorse. In both variable maps, all domains of prediction have easier and more challenging items to endorse, as was intended. Clearly shown, in the initial 81-item *PRE-Q* Wright map, is the considerable item logit-measure redundancy in the middle of the map (between +/- 0.50 logits). Redundant items (i.e., items with similar difficulty within equivalent predictive processing domains) were identified, and then item statistics and interpretation of item meaning by the panel members were used to select items for removal. After numerous iterations of item removal, the final parsimonious 19-item *PRE-Q* was established.

### Relationship to Other Variables

#### *PRE-Q* Differences by Diagnosis

Independent samples *t*-test findings revealed statistically significant differences in *PRE-Q* logit scores depending on whether the participant was neurotypical (*n* = 66, *M* = 1.07 logits, *SD* = 0.68 logits) or autistic (*n* = 59, *M* = 0.13 logits, *SD* = 0.93 logits); *t*(123) = 6.43, *p* < 0.001. The effect size was large (*η*^2^ = 0.252) with 25.2% in the *PRE-Q* logit score variance attributed to participant autistic diagnosis affiliation. These findings align with what was hypothesized.

#### Correlation between *PRE-Q* and Behavioral Task Performance Scores

Pearson correlation findings showed statistically significant moderate relationships between *PRE-Q* logit scores and two of the three behavioral task outcome measures (tapping imprecision and tapping correction (Task 2), but not timing sensitivity (Task 1)), among the full subsample of survey participants who completed the behavioral measures (*N* = 43: NT = 26, Autistic = 17). For the timing sensitivity measure (Task 1), there was no significant relationship between the outcome measure and the *PRE-Q* logit score; *r* (41) = 0.034, *p* = 0.83 (Figure S2 in the Supplemental Materials). When timing sensitivity of the two participant groups were examined separately, opposing effects were found. The autism group had a positive correlation between timing sensitivity and *PRE-Q* logit scores (*r* (15) = 0.384, *p* = 0.13), meaning that autistic adults with higher levels of self-reported prediction skills showed greater perceptual sensitivity, as expected. In contrast, the NT group unexpectedly had a negative correlation between timing sensitivity and *PRE-Q* logit scores (*r* (24) = -0.407, *p* < 0.05), meaning that NT adults who were less sensitive to perceptual shifts had a higher level of self-reported prediction skills.

For the tapping imprecision measure (Task 2), there was a statistically significant moderate negative relationship between imprecision and the *PRE-Q* logit score; *r* (41) = -0.368, *p* < 0.05, indicating that a greater precision in regular tapping was associated with higher prediction skills as measured by the *PRE-Q* (Figure S2 in the Supplemental Materials). The effect size was medium (*r*^2^ = 0.135), with 13.5% of variance in *PRE-Q* logit scores attributed to tapping imprecision. When the participant groups were examined separately, there was a significantly negative relationship between imprecision and the *PRE-Q* logit score for the autistic adults (*r* (15) = -0.624, *p* < 0.01), but not the NT adults (*r* (24) = -0.139, *p* = 0.50), suggesting that the relationship between tapping imprecision and *PRE-Q* logit scores was more related to differences in the autism group.

For the tapping correction measure (Task 2), the statistically significant moderate relationship was positive; *r* (41) = 0.398, *p* < 0.01, indicating that higher prediction skills as indicated by the *PRE-Q* logit scores was associated with better adaptation to a perturbed rhythm (Figure S2 in the Supplemental Materials). For this result, the effect size was also medium (*r*^2^ = 0.158) with 15.8% of *PRE-Q* logit score variance attributed to tapping correction. When the participant groups were examined separately, both the autistic and NT participants had similar positive correlation values between tapping correction and *PRE-Q* logit score (autistic: (*r* (15) = 0.398, *p* = 0.14; NT: (*r* (24) = 0.351, *p* = 0.08), suggesting that this outcome measure was related to self-reported prediction abilities for all participants.

## Discussion

### Evaluation of the *PRE-Q* Measure’s Performance

Overall, converging validity evidence suggests that the final 19-item *PRE-Q* has an effective rating scale, serves as a unidimensional and parsimonious measure of prediction-related daily experiences, and aligns with proposals of prediction-related challenges in autism. First, the 4-point rating scale effectively results in strong measure stability, accuracy, description of the sample, and inferences for the next sample (Linacre, 2002a). Second, the Rasch psychometric indices (infit, outfit, point-biserial correlation), measure consistency statistics (item and person reliability and separation), and item redundancy (logit-measures with *SEM* and variable map) indicated that the *PRE-Q* measures a unidimensional construct. Variable mapping revealed that the person and the item mean were similar, suggesting that the measure includes items that range across a hierarchy of difficulty. Third, the removal of redundant items of the questionnaire led to a parsimonious 19-item *PRE-Q*. Fourth, consistent with theoretical accounts of prediction-related challenges in autism, variable mapping revealed that the autistic participants tended to be clustered on the lower end of the distribution, *PRE-Q* logit scores differed significantly by diagnosis, and there were moderate correlations between the *PRE-Q* logit scores and two out of three behavioral measures of prediction (tapping imprecision, tapping correction) for the groups combined, and between the *PRE-Q* logit scores and all three behavioral measures for the autism group separately. Both the autistic and NT groups had similar correlation values between tapping correction and *PRE-Q* logit scores, indicating that the self-reported prediction skills were relevant to this prediction-relevant performance broadly across populations. Taken together, scores on the *PRE-Q* can be used as a composite measure of an individual’s self-reported prediction-related abilities. These results represent the first evidence showing a relationship between self-report and direct measurements of prediction-related auditory-motor tasks.

Importantly, the methods used for survey development incorporated both qualitative and quantitative information. The inclusion of comprehension and experience-relevance response options (i.e., “Did not understand the question” and “Did not have this experience”) allowed for incorporation of this qualitative information into the item development process. At each phase of data collection, items were re-worded to improve comprehensibility and to consider the reported universality of experiences of each item. During the evaluation process, ideas for new items from autistic individuals were solicited and incorporated, consistent with a participatory approach (Pickard et al., 2022). These responses were collected via optional online open-text questions; structured interviews may have yielded more nuanced information.

### Implications for Autism

Overall, the results from the *PRE-Q* align with the proposed frameworks of reduced prediction abilities of autistic individuals (Lawson et al., 2014; Pellicano & Burr, 2012; Sinha et al., 2014; van de Cruys et al., 2014). The significant correlations between the *PRE-Q* scores in the autism group and all three prediction-related behavioral measures (timing perception, tapping imprecision, tapping correction) provide additional evidence that the measure is relevant to auditory-motor prediction for autistic adults. Given this relevance, the domain-based content of the *PRE-Q* may guide clinicians in developing novel treatment approaches to better support and empower autistic individuals. Importantly, however, the *PRE-Q* is not currently validated for sensitivity to changes over time or for the use of domain-specific sub-scores independently from the composite score. If clinicians use the *PRE-Q*, they should also consider the potential overlap of scores on the *PRE-Q* with other cognitive processes including executive functioning. Additional investigation of the relationship between the *PRE-Q* and other measures of autism-related characteristics (e.g., The Comprehensive Autistic Trait Inventory (English et al., 2021), would provide insight into the relationship between these constructs.

Related to the domains of prediction, the item hierarchy revealed that items in the Social domain were generally more challenging for participants to endorse, while the items in the Motor and Sensory domains tended to fall in the middle and lower end of the difficulty range. Socially relevant items may be more difficult due to their highly dynamic nature. Additionally, given the particular relevance of the Social domain to characteristics of autism (American Psychiatric Association, 2013), the autism group may have influenced the overall distribution of these items towards the higher difficulty end by indicating greater challenges with the items in the Social domain. The domain-specific difficulty hierarchy revealed by autistic and NT participants may inform future conceptualization of prediction challenges and guide empirical research design (e.g., not assuming an equal level of difficulty across all domains). These results are consistent with studies in which autistic participants demonstrate domain-specific prediction differences with social stimuli (D’Mello et al., 2023). Clinicians who administer the *PRE-Q* should consider the distribution of items along the difficulty continuum when interpreting individual results.

### Limitations

The construct of prediction overlaps with other cognitive, social, attentional, and sensorimotor processes; these domains may therefore influence responses to items on the *PRE-Q*. For example, prediction may be closely related to executive functioning, working memory, or other constructs that were not measured in conjunction with the prediction questionnaire and whose relationship with prediction are not yet well established in the literature. Due to a lack of previously developed self-report or standardized behavioral measure of prediction, concurrent construct validity of the *PRE-Q* measure could not be analyzed in relation to any existing standardized data or measures.

Additionally, bias may have been introduced into the instrument at multiple stages of the development process. While the development team aimed to objectively represent the diversity and nuance of the prediction construct, there may be unaddressed bias in selection of items and content that align with their own proposed framework. As stated in the methods, the item development panel included a majority of individuals (9/10) from the same institution, including three individuals who were authors of a paper proposing prediction challenges in ASD (Sinha et al., 2014). The knowledge contributed by these subject matter experts enriched the development of a precise yet comprehensive construct definition, however, their presence on the measure development team may also pose a potential limitation in objectivity given the team’s investment in prediction as a valid and measurable construct as defined in several previously authored manuscripts. Notably, the author team also included two researchers who were affiliated with a separate institution and not involved with any work on the prediction framework prior to this project; their participation provided additional objectivity in the survey development process.

Bias may also have been introduced at the stage of field testing. Social Desirability Bias (Fisher & Katz, 2000), cultural values, and other factors such as self-awareness may have impacted how accurately individuals responded to items. In particular, autistic adults may demonstrate differences in self-awareness when compared to NT peers (Huang et al., 2017; Mazefsky et al., 2011). Lastly, there may be bias in who is represented in the sample. Because participants’ race and ethnicity data were not obtained, we are unable to determine whether the *PRE-Q’s* validity and relevance generalizes to the wider overall population. Future studies should include well-characterized, representative samples that report race, gender, educational attainment, and socioeconomic status to ensure generalizability.

## Conclusions and Future Directions

The present study provides converging evidence that the *PRE-Q* measures the day-to-day prediction skills of autistic and non-autistic adults. Consistent with theoretical accounts, autistic adults self-reported greater challenges with prediction-related tasks, and self-report of these difficulties was correlated with behavioral measures of auditory-motor prediction. Future research should validate the relationship between *PRE-Q* scores and additional empirical measures of prediction (e.g., across various domains and sensory modalities), evaluate its relevance to other populations for which prediction challenges have been proposed (e.g., schizophrenia, dementia), and investigate the relevance of the domain-specific sub-scores independent from the composite score. Building upon the initial validity evidence of the *PRE-Q* across research and clinical contexts can enrich the scientific understanding of the role of prediction in autism and may help to better characterize and reduce prediction-related challenges experienced by autistic adults.

## Footnotes

1 Many, but not all, autistic individuals prefer the use of identity-first language (“autistic adult”) over person-first language (“adult with autism”; Taboas et al., 2023). These preferences guide the authors’ language choices in the present article, consistent with American Psychological Association style guidelines related to the use of bias-free, inclusive language (American Psychological Association, 2022).
